# Microstructure study of a severely plastically deformed Mg-Zn-Y alloy by application of low angle annular dark field diffraction contrast imaging

**DOI:** 10.1080/14686996.2016.1140304

**Published:** 2016-04-11

**Authors:** Dudekula Althaf Basha, Julian M. Rosalie, Hidetoshi Somekawa, Takashi Miyawaki, Alok Singh, Koichi Tsuchiya

**Affiliations:** ^a^ Structural Materials Unit, National Institute for Materials Science, Sengen 1-2-1, Tsukuba, 305-0047Japan.; ^b^ Graduate School of Pure and Applied Sciences, University of Tsukuba, Tsukuba, Ibaraki, 305-8577Japan.; ^c^ Erich Schmid Institute of Materials Science, Austrian Academy of Sciences, Austria.

**Keywords:** STEM, LAADF, diffraction contrast, severe plastic deformation (SPD), recrystallization, high pressure torsion (HPT), 10 Engineering and structural materials, 503TEM, STEM, SEM, 106 Metallic materials

## Abstract

Microstructural investigation of extremely strained samples, such as severely plastically deformed (SPD) materials, by using conventional transmission electron microscopy techniques is very challenging due to strong image contrast resulting from the high defect density. In this study, low angle annular dark field (LAADF) imaging mode of scanning transmission electron microscope (STEM) has been applied to study the microstructure of a Mg-3Zn-0.5Y (at%) alloy processed by high pressure torsion (HPT). LAADF imaging advantages for observation of twinning, grain fragmentation, nucleation of recrystallized grains and precipitation on second phase particles in the alloy processed by HPT are highlighted. By using STEM-LAADF imaging with a range of incident angles, various microstructural features have been imaged, such as nanoscale subgrain structure and recrystallization nucleation even from the thicker region of the highly strained matrix. It is shown that nucleation of recrystallized grains starts at a strain level of revolution N=1/4 (earlier than detected by conventional bright field imaging). Occurrence of recrystallization of grains by nucleating heterogeneously on quasicrystalline particles is also confirmed. Minimizing all strain effects by LAADF imaging facilitated grain size measurement of 150±25 nm in fully recrystallized HPT specimen after N=5.

## Graphical Abstract

**Figure:**
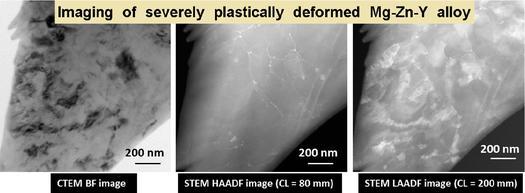

## 1. Introduction

In recent years severe plastic deformation (SPD) has been commonly employed to improve the mechanical properties, e.g. strength, ductility and toughness, of metallic materials. Mechanical properties improve because of grain refinement by dynamic recrystallization processes and strain accumulation. Submicron size grain can easily be achieved by SPD processes. SPD also modifies the crystallographic texture of the alloys, which can have direct impact on the mechanical (deformation) behavior. Some of the common SPD techniques include repeated rolling, such as equal channel angular extrusion (ECAE) [1], and caliber rolling [2] and high pressure torsion (HPT) [3].

In order to understand the deformation and recrystallization process during SPD, as well as the mechanical properties, it is important to study the microstructure such as grain structure and phase distribution, and deformation structures such as dislocations and twins. Because of the fine scale of the structures, as well as to obtain crystallographic information, it is necessary to use electron microscopy, especially transmission electron microscopy (TEM). However, the severely deformed microstructures are a challenge for conventional TEM (CTEM), which works well with defect-free annealed samples or with discrete lattice defects. Strain introduced by severe plastic deformation makes it difficult to look past the strong diffraction contrast.

STEM is a powerful imaging mode for detailed analysis of crystalline materials and for investigating electron beam-specimen interactions [4–8]. STEM has a considerable advantage over conventional TEM because various signals emitted from the same specimen can be collected simultaneously by utilizing different detectors having different shapes and sizes and thus effectively used for efficient imaging with high contrast. High angle annular dark field (HAADF) imaging is an important tool in STEM mode. HAADF images are formed by incoherent thermal diffuse scattering (TDS) electrons, utilizing a large inner angle detector [4,9]. Furthermore, in incoherent imaging, each atom independently contributes intensity to the resulting Z-contrast image. Howie suggested the use of an annular detector with the inner collection angle exceeding 40 mrads to ensure that all the scattering was thermally diffused incoherent scattering [9]. Apart from studies over several years using Z-contrast imaging techniques,diffraction-contrast STEM imaging methods have recently found much interest in the study of crystal defects, which is a major branch of CTEM [10–12]. If the inner detection angle of the annular dark field (ADF) detector is small, Bragg reflections from crystalline materials can significantly contribute to the ADF-STEM imaging. As the inner collection angle decreases, the scattering from crystals is no longer incoherent in nature, as other effects such as low-angle scattering from precipitates occur. Contrast reversal effects contribute to ADF imaging. At low scattering angles the diffraction contrast effectively becomes stronger than Z contrast and the imaging will become more similar to conventional dark-field TEM imaging. STEM imaging of crystalline defects can offer several practical benefits that are impossible to realize in CTEM imaging. It has been shown that STEM imaging allows for more uniform contrast of large sample areas with reduced thickness fringes, bend contours, and other auxiliary contrast effects and with greater signal-to-noise ratio and precisely resolved defects even in thick specimens which are difficult to resolve in traditional CTEM imaging [10]. Conventional techniques that are applicable in CTEM for the imaging of crystal defects, such as finding the Burgers vector of a dislocation or the displacement vector of a stacking fault, are also applicable in STEM [13].

**Figure 1.**
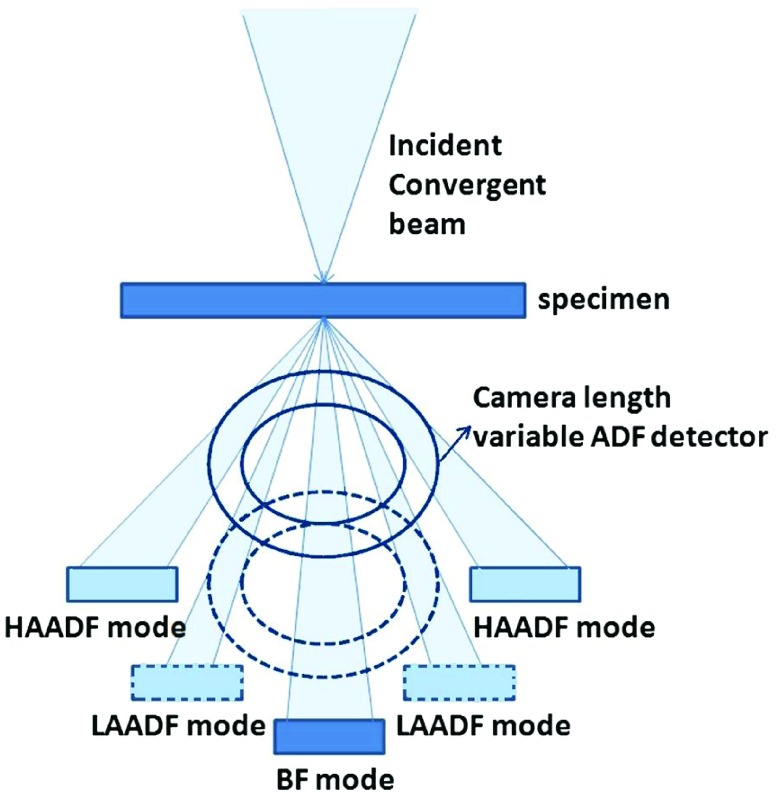
Schematic diagram of the virtual displacement of the ADF detector in STEM mode. The ADF detector is at the upper position in HAADF mode to collect high angle scattered electrons which contain Z-contrast information, and at the lower position in LAADF mode to collect low angle scattered electrons which contain both Z-contrast and diffraction contrast information. The unscattered rays can be collected by the BF detector.

Figure 1 shows a schematic diagram of STEM with BF and the ADF signal detection geometry. The bright field (BF) STEM image is formed by collecting the transmitted signal and it appears similar to the CTEM phase contrast image. The ADF detector is centered with respect to the optic axis of the microscope and the scattered electrons over an angular range can be collected by virtually (electronically) shifting the detector position along the optic axis with respect to sample, i.e. by changing the camera length [14]. The ADF detector is in the upper position in HAADF mode to collect high angle scattered electrons which contain Z-contrast information, and it is at the lower position in LAADF mode to collect low angle scattered electrons which contain both Z-contrast and diffraction contrast information. In general, the strength of the magnetic lenses is varied accordingly to project high- or low-angle scattered electrons onto the fixed annual detector. The displacement of the detector position in STEM mode corresponds to the beam tilt in CTEM. The image contrast is sensitive to the displacement of the ADF detector and thus certain features of the specimen can be enhanced by collecting a specific portion of the scattered signals, a technique which is extremely useful for imaging inhomogeneous and deformed samples. The degree of coherence decreases as the scattered angle of electrons increases for both the elastic and the inelastic imaging of thin objects.

In this work, for the first time, STEM-LAADF methodology has been applied to observe the overall deformation and recrystallization of grains by HPT. In the HPT process, a disk shaped sample is compressed between two dies under a high compressive stress of a few GPa, and then one of the dies is given a rotation to create torsion. The applied strain is proportional to the number of rotations, given by $\varepsilon =\frac{\pi Nr}{\sqrt{3}t}$, where ε is the strain, *N* is the number of turns, *r* is the distance to the disk center and *t* is the sample thickness. In HPT a high strain can be applied even at low temperatures, such as room temperature, because the physical constraint on the sample by the dies does not let the sample break.

STEM imaging within a particular angular range or with a certain camera length to observe grain fragmentation and recrystallization initiation in severely plastically deformed microstructures is demonstrated with a number of illustrations. Although the principles of this kind of imaging are well known, they have not been covered by any specific theoretical paper in detail. It has also been shown that LAADF imaging would be a uniquely powerful tool to observe the sharp twin boundary or grain boundary contrast in deformed recrystallized regions of the specimen, and to estimate accurate recrystallized ultrafine grain size, especially in specimens containing secondary phase particles of similar size as that of the grains. The alloy studied here is a Mg-3Zn-0.5Y (at%) alloy, in which Mg-Zn precipitates create a distinct compositional contrast in the magnesium matrix. Yttrium-bearing ternary phases (mainly quasicrystalline icosahedral phase) display a higher level of compositional contrast. Thus the samples of this alloy provide diffraction as well as compositional contrast. In this alloy composition, when processed by extrusion, very high mechanical strength accompanied by ductility has been reported [15]. The strengthening and ductility is attributed to fine grain size obtained by dynamic recrystallization during extrusion and simultaneous modification of texture, aided by presence of the quasicrystalline phase. Thus the main interest of this study is observation of recrystallization by introduction of different amounts of strain by means of HPT. In an earlier work, we have also studied Mg-3.4at%Zn binary alloy processed by HPT [16,17]. In this study, early recrystallization of grains has been detected by imaging with LAADF. Segregation of zinc occurs on newly created boundaries. Grain boundaries of recrystallized grains have been distinguished by creating different contrasts. A systematic application of these imaging techniques to SPD alloys has been shown for the first time.

## 2. Experimental details

An alloy of nominal composition Mg-3Zn-0.5Y (at%) was prepared by melting pure metals under protective atmosphere in an electric furnace and cast in a steel mold. The cast material was extruded and then subjected to caliber rolling (eight passes) to form into a rod. The rod was annealed at 573 K for 24 h in order to anneal out the strain due to rolling, for grain growth and for dissolution of the Mg-Zn binary precipitates. After annealing, a grain size of about 10 μm was obtained. The annealed rods were machined into rods of 10 mm diameter, from which circular disks of 1 mm thickness were cut. These disks were ground down to 0.85 mm thickness and subjected to high pressure torsion (HPT). The HPT processing was performed at room temperature at a constant pressure of 5 GPa in which the lower anvil was rotated at a speed of 1 rpm. Disks were deformed through a total number of revolutions, *N*, of 0, 1/4, 1/2, 1 and 5 turns. Of these, results of only samples of N=0, 1/4 and 5 are described, because of the significant changes in them. For microstructure characterization, samples were carefully extracted at a distance of 2–3 mm from the center of the disk.

**Table 1.** ADF detector acceptance angles as a function of camera length.

	**ADF detector acceptance angle (mrad)**	
**Camera length (mm)**	Inner, $\theta_{i}$	Outer, $\theta_{o}$
80	50.0	336.6
100	40.0	273.0
120	33.3	233.3
150	26.6	184.4
200	20.0	139.0
250	16.0	111.5
300	13.3	93.0
380	10.5	73.5
560	7.1	50.0
1200	3.3	23.3

TEM samples were prepared by ion milling (Gatan, model 691, Gatan Inc, Pleasanton CA, USA) after mechanical grinding and then examined using a FEI-Tecnai F30 (FEI Technologies Inc., Hillsboro, Oregon, USA) microscope operating at 300 kV. Microstructure observations were performed both in conventional CTEM and in STEM mode with a probe size of 2 nm. Energy dispersive X-ray spectroscopy (EDS) was performed in STEM mode. STEM-HAADF images were recorded by Fischione model 3000 HAADF detector (E.A. Fischione Instruments Inc., PA USA) using camera lengths ranging from 560 to 80 mm (occasionally 1200 mm), corresponding to the inner collection angle in the range of few mrad to 50 mrad. The inner and outer diameters of the ADF detector were 4 and 28 mm, respectively, and the beam convergence angle was 18.6 mrad. Table 1 shows a list of camera lengths for the Tecnai F30 microscope and corresponding acceptance angles. CTEM was also performed on a JEOL 2000FX microscope (JEOL Ltd., Tokyo, Japan).

For convenience, change in image contrast is represented with respect to change in camera length, instead of scattering angles, since each camera length of detector needs to be represented by two angles, the inner and outer acceptance angles. Based on experimental observations of diffraction effects, micrographs recorded with camera lengths of 80 mm are termed HAADF-STEM images, whereas those with camera lengths greater than 80 mm are termed LAADF images in this paper.

## 3. Results and discussion

Deformation by HPT was studied by observing samples with increasing strain from the compressive stress and N=0-5 turns of torsion. The deformation started with twinning accompanied by dislocation slip. Subsequently, concentration of stress can occur at specific locations such as twin boundaries, leading to initiation of recrystallization. Nucleation of recrystallized grains also occurs on second phase, such as quasicrystal phase in the present alloy.

**Figure 2.**
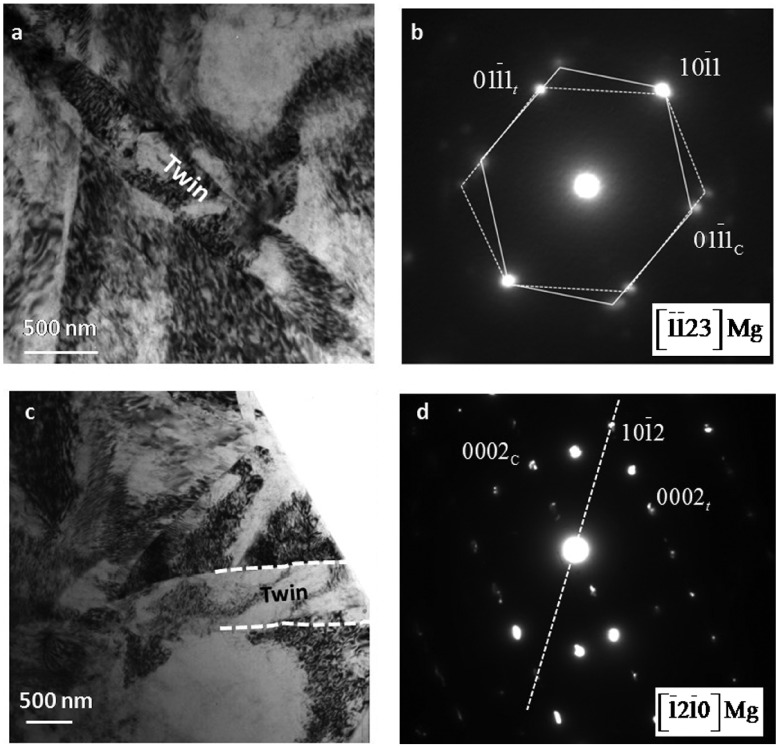
Conventional TEM bright field micrographs showing deformation twins in N=0 HPT sample of Mg-3Zn-0.5 (at%) alloy. (a) A twin viewed along [$\bar{1}\bar{1}$23] zone axis, and the corresponding composite diffraction pattern (b). The twin system is {10$\bar{1}$1}$\left\langle 10\bar{1}2\right\rangle$. (c) Another twin viewed along [12$\bar{1}$0] zone axis and the corresponding composite diffraction pattern (d). The twin system is $\left\{10\bar{1}2\right\}\left\langle 10\bar{1}1\right\rangle$.

### Twinning and dislocation slip in the N=0 sample, and the specimen tilt dependent channeling contrast

3.1. 

As is well known for hexagonal metals, wrought processing introduces a strong texture, in which the grains tend to align with basal planes in the direction of mechanical working (such as rolling or extrusion). Hexagonal metals commonly tend to twin by $\left\{10\bar{1}2\right\}$ type of twinning (extension twin) when a tensile stress is applied along the hexagonal axis [18,19]. Such a situation occurs in the experimental setup of the present study. The disks subjected to HPT are sliced in cross-section from a rolled bar, in which grains are expected to be aligned preferentially with their basal planes along the compression axis. Therefore, massive twinning occurs on application of compressive pressure, observed in the N=0 sample.

Figure 2a and c show BF-TEM images of the two types of deformation twins in N=0 HPT (compressed) sample. The images are viewed along [$\bar{1}\bar{1}$23] and [$\bar{1}2\bar{1}0$] zone axis, respectively. The mirror images of diffraction spots with respect to {10$\bar{1}$1} and {10$\bar{1}$2} planes in selected area diffraction patterns (SADP) of Figure 2b and d clearly show the type of twins. Twinning under these conditions, characterized by {10$\bar{1}$1} and {10$\bar{1}$2} twin planes, cause a lattice reorientation of approximately 56${}^{\circ }\;$and 86${}^{\circ }\;$, respectively, around a $\left\langle 11\bar{2}0\right\rangle$ axis. In addition, strong strain contrasts are observed in these micrographs, which arise due to a high density of dislocations. These dislocations have evidently formed due to the high amount of stress of 5 GPa, as well as easy slip caused by reorientation of lattice due to twinning. For this reason of high strain, twin boundaries do not appear very well defined. {10$\bar{1}$1} type twins are usually known to form at a high level of stress.

**Figure 3.**
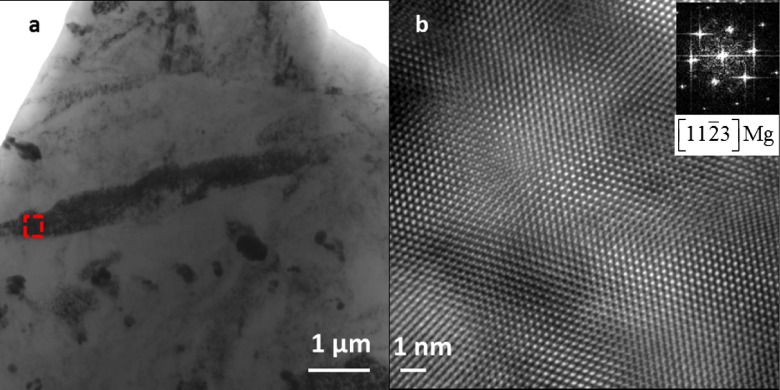
(a) Conventional TEM bright field image showing a twin oriented along [11$\bar{2}$3] zone axis. (b) HRTEM image with FFT (inset) of the same twin acquired from a small region shown in square box in (a).

Next, the contrast of twins under HAADF-LAADF is examined. In the present investigations, channeling effects need to be avoided systematically, since they can prevent a direct interpretation of the HAADF and LAADF images in STEM mode in terms of chemical localization by Z-contrast and Bragg diffraction contrast respectively. Figure 3 shows BF-TEM and high resolution TEM (HRTEM) images of a typical twin found in the N=0 HPT sample. The twin plate is oriented along the $\left[11\bar{2}3\right]$ zone axis as shown in the HRTEM image and its fast Fourier transform (FFT) inset. Local variations in lattice spot intensities indicates strain in the lattice

**Figure 4.**
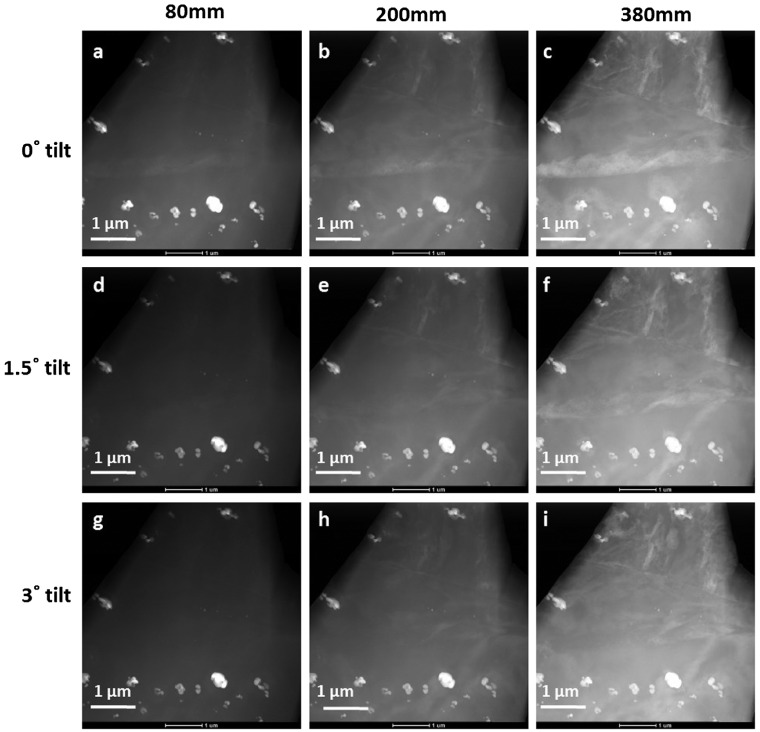
A series of STEM-ADF images showing variation of twin contrast from the same region as shown in Figure 3a, at different tilt positions and at different camera lengths. Rows from top to bottom: tilts of 0${}^{\circ }\;$, 1.5${}^{\circ }\;$and 3${}^{\circ }\;$, columns from left to right: camera lengths 80, 200 and 380 mm.

This sample region was then examined in ADF mode, as shown in Figure 4. The sample was tilted in STEM mode by about 0${}^{\circ }\;$, 1.5${}^{\circ }\;$and 3${}^{\circ }\;$away from the zone axis, and the effect of tilt on imaging was observed. At each tilting position, ADF images of the same twin were acquired with camera lengths of 80, 200 and 380 mm. It is assumed that upon tilting the sample a few degrees away from the zone axis, thickness remains unchanged, as ADF image intensity depends strongly on the mass thickness of the sample. When the sample is at 0${}^{\circ }\;$tilt, the HAADF image acquired with a camera length CL = 80 mm image (Figure 4a) shows bright contrast not only from the ternary phase particles due to Z-contrast, but also from twin plate due to channeling effects along the zone axis. In the same tilt conditions, LAADF images acquired with CL = 200 and 380 mm (Figure 4b and c) show much stronger bright contrast from the twin due to additional contributions from diffracted beams as well as channeling effects along the zone axis. These LAADF images still exhibit bright contrast from the particles as the Z-contrast contribution is still retained. When the sample is tilted 1.5${}^{\circ }\;$away from the zone axis, the twin intensity in HAADF image (Figure 4d) decreases significantly and does not exhibit any bright contrast, whereas the twin still exhibits a reasonable intensity in LAADF images (Figure 4e and f). At further tilt to 3${}^{\circ }\;$, the twin does not show any bright contrast in either HAADF (Figure 4g) or LAADF (Figures 4h and i) images (while other strain contrast are visible). In general, the HAADF image (Figure 4a) should not exhibit any bright contrast since at high scattering angles only Z-contrast will be expected. This demonstrates the contribution of channeling effects from well-aligned atomic columns in the zone axis oriented twin. These channeling effects will not be seen when the crystal is tilted even a few degrees away from the zone axis and hence the twin in the HAADF image (Figure 4d) loses its intensity by tilting just 1.5${}^{\circ }\;$away from zone axis. The higher brightness contrast from the twin in the LAADF image (Figure 4c) compared to the HAADF image (Figure 4a) indicates the strong dominance of those low order diffraction disks that fall on the ADF detector. At the same time, it is not possible to disregard the contributions from channeling effects at low scattering angles since twin intensity in the LAADF image (Figure 4e and f) slightly decreases when the twin is tilted 1.5${}^{\circ }\;$from the exact zone and it completely loses its intensity with an additional tilt of 3${}^{\circ }\;$from the zone axis (Figure 4h and i). The existence of weakly bright intensity of the twin in the LAADF image (Figure 4e and f) when it is in a 1.5${}^{\circ }\;$tilt condition from low index zone shows that the contribution of diffraction effects is not as sensitive as that of the channeling effects to the zone axis. From the above observations it can be seen that contributions of intensities for HAADF and LAADF imaging are not from the same source and that by tilting the sample and/or by changing the camera length, the source of contrast can be varied dramatically. These systematic tilting studies in HAADF and LAADF modes eliminate the possibility of presence of any recrystallized grains at the strain level in the N=0 HPT specimen.

**Figure 5.**
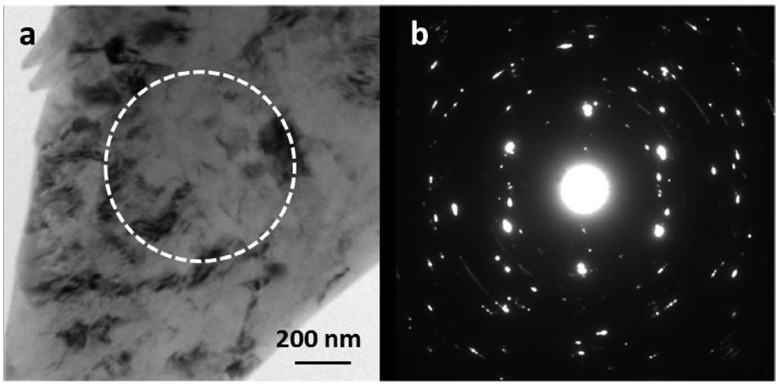
(a) Conventional TEM image recorded from a deformed region of N=1/4 HPT processed sample. (b) SADP acquired from corresponding region marked in (a), depicting multiple diffraction spots and arcs, which indicate the formation of low angle boundaries.

### 3.2. Subgrain structure formation in N=1/4 sample

**Figure 6.**
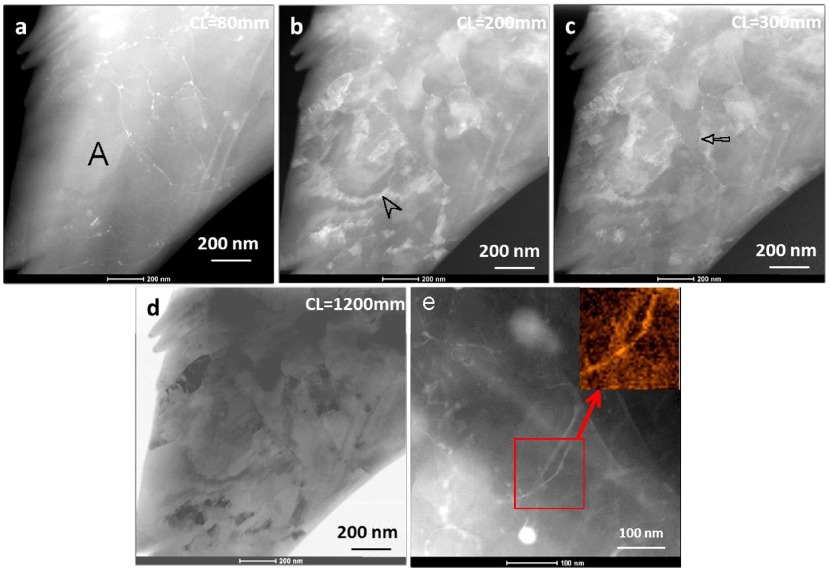
STEM-ADF images acquired from the same area as shown in Figure 5, in N=1/4 HPT processed sample. (a) HAADF image (CL = 80 mm) showing only Zn segregation on low angle boundaries. (b–d) LAADF images (CL = 200, 300 and 1200 mm) exhibiting varying contrast from individual fragmented grains clearly, which is not observed either in CTEM or HAADF images. Furthermore, the STEM-ADF acquired with large camera length of 1200 mm (d) resembles an annular bright field (ABF) image. (e) STEM-ADF image from another region in the sample. The EDS map of zinc from an area marked by a square is shown in the inset.

Further slip of dislocations occurs when torsion is applied to the sample, in addition to the 5 GPa load. This leads to formation of subgrains, which occurs when dislocations align to form subgrain boundaries. Figure 5 shows the microstructure of one of the regions of the N=1/4 sample. At this strain level, strain contrast at a fine scale is discerned in the CTEM image in Figure 5a. The accompanying diffraction pattern in Figure 5b shows spots belonging to the $\left[11\bar{2}0\right]$ zone axis, and a number of other spots indicative of grain fragmentation. However, it is difficult to observe grain fragmentation or formation of subgrain structures in the bright field image due to the high accumulated strain. The HPT deformation process with repeated revolutions leads to generation of excessive dislocations in grain interiors, which further leads to formation of dislocation cell structures and subgrains with low angle grain boundaries. Therefore the same region as shown in Figure 5 was imaged using STEM-ADF with several different camera lengths, shown in Figure 6. The HAADF image acquired with CL = 80 mm (Figure 6a) shows lines of Zn enrichment, which indicate formation of new boundaries, possibly low angle grain boundaries. Enrichment of zinc on boundaries by possible segregation of zinc is confirmed by EDS mapping shown in Figure 6e. In Figure 6a–d, a large region in the middle-left of the micrograph, marked ‘A’ in (a), does not show similar boundaries. In the LAADF images of Figure 6b acquired with higher camera lengths of 200 mm, regions of strain can be observed, which are seen to terminate at the boundaries. In the larger region A in the middle-left, there is a large ring of continuous strain contrast, indicated by an arrowhead, which suggests that this region is a single large grain containing lattice strain. On increasing the camera length to 300 mm (Figure 6c), the contrast becomes softer, and more subtle details become visible. More well-defined subgrains are observed, such as one marked by an arrow. The STEM-ADF image acquired with CL = 1200 mm (Figure 6d) appears similar to an annular bright field (ABF) image due to contributions from the peripheral transmitted beam at very high camera lengths [20]. By appearance it corresponds to the ADF image of Figure 6b. This example shows that STEM-ADF imaging becomes invaluable in observation of early deformation pathways before recrystallization occurs.

### 3.3. Imaging of recrystallized grains nucleated in thick and highly strained region

**Figure 7.**
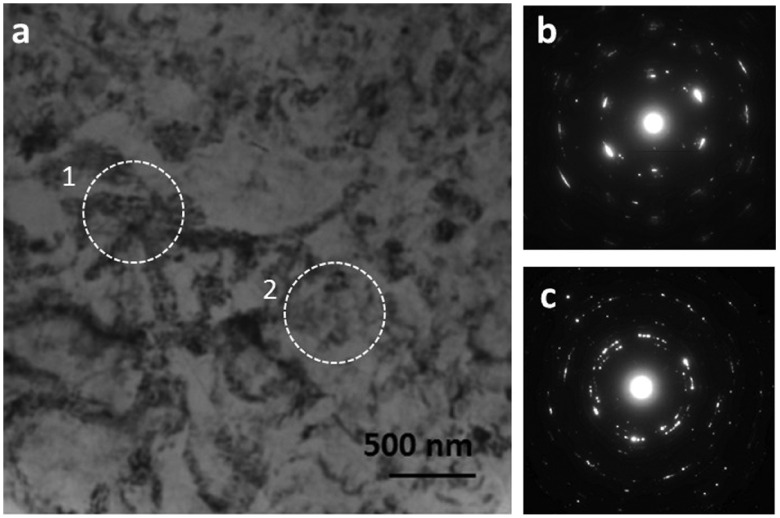
(a) Conventional TEM bright field image acquired from highly strained region of N=1/4 HPT sample. (b) Spreading arc spots in SADP acquired from region 1 in (a), indicating an unrecrystallized region. (c) Concentric rings in SADP acquired from region 2 in (a), indicating recrystallization in that region even though recrystallized grains are not seen.

**Figure 8.**
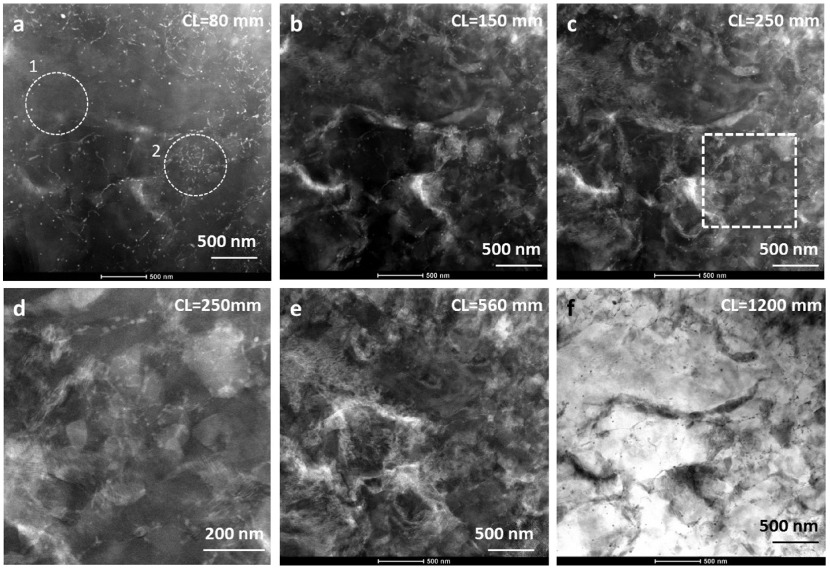
STEM-ADF images acquired from the same area as shown in Figure 7a in the N=1/4 HPT sample. (a) HAADF image acquired with camera length 80 mm showing Z-contrast information. (b) LAADF image acquired with camera length of 150 mm showing not only Z-contrast information but also exhibiting varying contrast from lattice strain owing to diffraction contrast. (c) LAADF image acquired at camera length of 250 mm, additionally exhibiting a varying contrast from few recrystallized grains (shown in box) from high angle boundaries, clearly owing to a diffraction contrast not observed in either CTEM or HAADF images. The boxed region is shown magnified in (d). (e–f) STEM-ADF acquired with large camera lengths of 560 and 1200 mm, again exhibiting contrast only from lattice strain. This indicates that the visibility of grains depends on proper choice of camera length for a particular beam convergence angle and specimen thickness.

Figure 7a shows a CTEM dark field micrograph at another location in the N=1/4 sample. Regions of strain concentration are imaged, which indicate local lattice distortion in Bragg condition. No grain boundaries are visible. Specimen thickness in this region was determined to be 226 nm by measuring fringe width in a disk of {10$\bar{1}$0} convergent beam electron diffraction pattern in a two-beam condition. Two regions, 1 and 2, are marked by circles. The diffraction pattern acquired from region 1, shown in Figure 7b, exhibits diffraction spots from essentially the same zone axis, though the spots are in the form of arcs indicative of strain. This suggests that this region is still unrecrystallized. The diffraction pattern shown in Figure 7c, acquired from region 2, exhibits concentric rings, which suggest the presence of several grains in this small region, i.e. the formation of recrystallized grains (having large angle grain boundaries). Since the CTEM image does not show any recrystallized grains, further investigation was carried out by STEM-ADF imaging. Figure 8 shows a series of corresponding STEM-ADF micrographs acquired from the same region of the sample with different camera lengths. The HAADF image acquired with CL = 80 mm under the same sample tilt conditions as in Figure 7 is shown in Figure 8a. A distribution of sharp bright contrast appears, which can be attributed to boundaries decorated with segregation of zinc. The contrast of this region is changed quite dramatically in the LAADF image acquired with a camera length of 150 mm, shown in Figure 8b. Now strain contrast is the dominant feature, although it still shows the Z-contrast information. This contrast obscures any detailed information. With further increase in the camera length, Figure 8c, the strain contrast is softened and more details are visible in between. Figure 8d shows a magnified view of the square box region in Figure 8c, which corresponds to region 2 in Figure 7a. In this micrograph, grains with a size of about 100 nm with sharp grain boundaries are clearly observed. The distinction between regions 1 and 2 of Figure 7a, unrecrystallized versus recrystallized, is also confirmed from Figure 8a, in which region 1 does not show presence of any grain boundaries by zinc segregation contrast, while region 2 shows a high density of grain boundaries decorated with zinc contrast. However, grain boundaries can be observed such as in Figure 8d even in the absence of any Z-contrast. This example demonstrates that ADF imaging with conical illumination, using an appropriate camera length for the ADF detector to remove the strain effects, becomes extremely useful to retrieve a large amount of embedded structural information. Information from such buried structures is more difficult to access with traditional TEM imaging, leading to a quick and incorrect conclusion that recrystallization nucleation has not yet occurred. Furthermore, the visibility of recrystallized grains depends on beam convergence angle, camera length and specimen thickness. In this example the sample thickness of about 200 nm is comparable to the scale of the microstructure in which the grain size is in the range of 100–200 nm. This illustration shows the power of low-angular filtered ADF imaging as a unique tool for observing recrystallized grains and their formation mechanisms from very interior locations of the deformed sample at early periods of applied strain. With further increase in the camera length, Figure 8e, the composition contrast is at a minimum, and finer details of strain contrast are visible. At camera length of 1200 mm, Figure 8f, the contrast looks like a reversal of CL = 150 mm (Figure 8b). The contrast is now equivalent of ABF.

### 3.4. DRX nucleation on second phase particles

**Figure 9.**
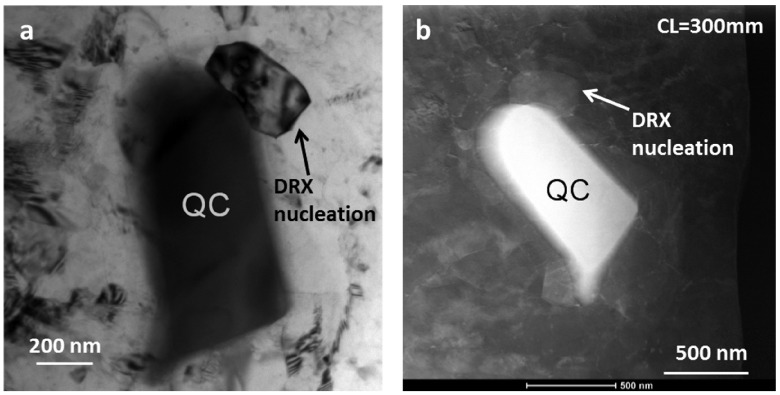
(a) Conventional TEM bright field image showing a DRX grain nucleated on Mg${}_{3}$Zn${}_{6}$Y quasicrystalline (QC) particle in N=1/2 HPT processed sample. (b) STEM-LAADF images (CL = 300 mm) showing diffraction contrast from newly recrystallized grains nucleated on the quasicrystalline particle.

Secondary phase particles of hard phases are known to promote dynamic recrystallization (DRX) [21]. Early recrystallization occurs over secondary phase particles. The effect of fine particles on DRX and grain refinement mechanisms is also one of the major points in investigation on severely deformed magnesium alloys. The case of the secondary phase being the quasicrystal phase is of particular interest, because recrystallization on it can modify the grain texture [15,22]. The icosahedral quasicrystal phase has point group symmetry of $m\bar{3}\bar{5}$, with a large order of symmetry of 120. Assumption of only a single orientation relationship of $2f_{ico}{\|\left(0001\right)}_{hex}$ [23] can produce 15 variants of a hexagonal crystal. More orientation relationships are known to occur between magnesium and quasicrystal phase [24]. Figure 9a shows a DRX grain attached to a quasicrystal phase particle. This grain appears to be free of severe strain and exhibits a sharp grain boundary, and is therefore assumed to be recrystallized, by nucleating on the quasicrystal particle. Other than this grain (and the quasicrystal particle), only strain contrast is visible in this micrograph. This region was then imaged in STEM-ADF mode. The HAADF image acquired with CL = 80 mm displays only bright contrast from the quasicrystal particle. Figure 9b shows a micrograph recorded with camera lengths of 300 mm, which reveals bright contrast from the recrystallized grain attached to the quasicrystal particle. In this image, more small grains are seen attached to the quasicrystal phase particle, having recrystallized by nucleating over the particle. Thus it is established that quasicrystal particles effectively act as heterogeneous nucleation sites for dynamically recrystallized grains, even in a sample with relatively low amount of strain. Diffraction patterns to confirm crystallographic relationship were obtained (not shown here).

### 3.5. Observation of grain boundaries of ultrafine nanosize grains in recrystallized regions

It is very important to determine and to control grain size and secondary phase particles or precipitates size, as these have a significant effect on the mechanical properties of metals and alloys. The size of the grains can vary in a wide range, from very few tens of nanometers to up to a size of 1 μm in ultrafine grain alloys. From traditional CTEM imaging it can be challenging to distinguish between a matrix grain and a second phase particle because of the presence of both mass-thickness contrast and diffraction contrast. Misidentification of grain with particle can lead to incorrect estimation of grain size or particle size distribution, determination of which is very important for microstructural characterization and consequently the optimization of mechanical properties. To avoid such complications, LAADF/HAADF coupled STEM imaging can be effectively utilized to distinguish grains, at first from strain contrast, and then from second phase particles, and then to measure their size distribution.

**Figure 10.**
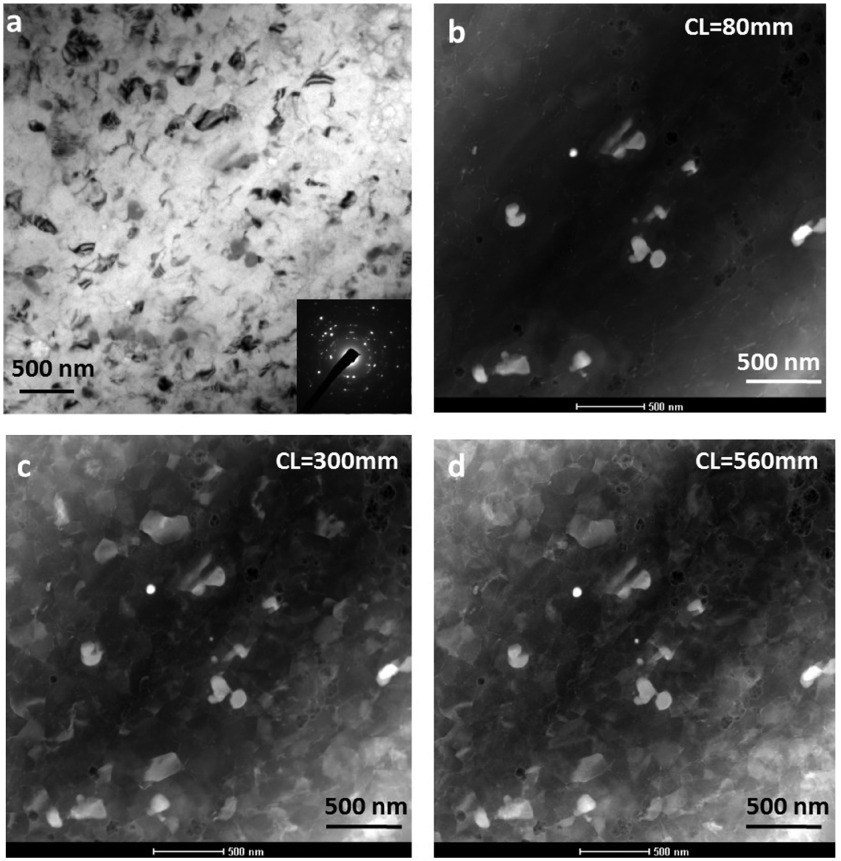
(a) Conventional TEM bright field image showing a recrystallized region in N=5 HPT sample. The concentric rings in SADP as shown in the inset indicate recrystallization occurrence. (b) HAADF image acquired from same region with camera length 80 mm showing only Z-contrast. (c) LAADF image acquired from same region with camera length 300 mm showing grain refinement. (d) LAADF image acquired with camera length 560 mm. Both HAADF and LAADF imaging techniques together can be used to find grain size distribution and particle size distribution.

**Figure 11.**
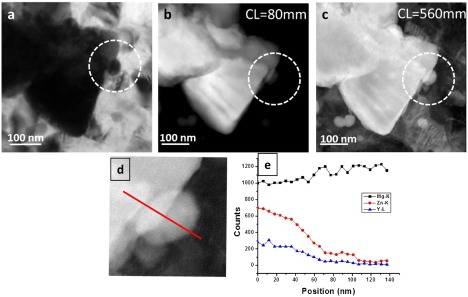
Observation of nucleation of a precipitate on a second phase particle. (a) Conventional TEM image showing a small precipitate seemingly attached to the second phase particle. (b) HAADF image (CL = 80 mm) acquired from the same location shows weak intensity from the precipitate. (c) LAADF image (CL = 380 mm) acquired from the same location shows bright intensity from the precipitate. (d) EDS line composition profile across the precipitate. Here both HAADF and LAADF imaging together confirm that this particle is a precipitate.

Figure 10a shows a CTEM bright field image containing recrystallized grains and secondary phase particles from a recrystallized region in the N=5 HPT sample. This image also shows that recrystallization has occurred over the entire region of the sample. However, the grains cannot be distinguished clearly, in part due to uneven strain contrast within grains and partly because of uneven thickness of the region. The sample is thinner in a diagonal region from the bottom left to top right. Figure 10b–d shows three corresponding ADF images from the same location with three different camera lengths of 80, 380 and 560 mm. The advantage of LAADF/HAADF STEM coupled imaging for identifying grains and particles can be clearly observed. The HAADF image acquired at the shorter camera length of 80 mm shows bright contrast from only secondary phase particles. These particles are indistinguishable from the matrix grains in the bright field image of Figure 10a. In LAADF images acquired at higher camera lengths of 300 and 560 mm, the individual grains and grain boundaries are also clearly visible apart from the secondary phase particles. From a number of HAADF/LAADF images acquired from different locations of the sample, by measuring the farthest distance between recrystallized grain corners from the LAADF images of over 250 grains, the average grain size after five rotations of HPT is found to be 150±25 nm. Thus, in this case both HAADF-STEM and LAADF-STEM investigations must be performed to gain information about grain size distribution and particle size distribution. Moreover, in the CTEM image (Figure 10a), only few grains show strong diffraction contrast, whereas in STEM-LAADF images (Figure 10c–d) acquired with CL = 300 and 560 mm more grains exhibit strong diffraction contrast. This is because the convergent beam used in STEM imaging facilitates the beam to incident on a sample over a range of angles, hence even the grains slightly away from Bragg condition with respect to the parallel beam also exhibit diffraction contrast. In the case of observation by CTEM, grain boundaries are not clearly visible, therefore dark field observation is employed to look at the grain size, such as in our previous report [16]. By this method, only a few grains are in contrast at any instance, and the grain boundaries are still not imaged due to strain contrast near the boundaries. Therefore the estimation of grain size is not accurate. In the micrographs of Figure 10, a qualitative difference in imaging between thin and thick regions can be observed. This is because, in general, in STEM-ADF (HAADF or LAADF) imaging, as the specimen thickness increases, the background intensity also increases due to the increase in elastic scattering. In LAADF imaging, the background intensity from thick regions of the specimen dominates. The diffracted beam intensities from thinner regions are weaker as compared to background intensity from thicker regions. It can be noticed in the micrographs that the contrast difference is reduced as the camera length increases.

Earlier study of Mg-Zn binary HPT alloy reported round particles of a Mg-Zn phase of about 10–20 nm in size at strain of N=20 [16]. Similar particles were also observed in this study. Figure 11a shows a CTEM bright field image of specimen N=5. Smaller dynamically nucleated precipitates are present in an Mg matrix. They are also observed on the ternary phase particle (yttrium rich) boundaries. From the CTEM image of the thick specimen, it is very difficult to identify whether a particular phase attached to it is a dynamically nucleated precipitate or broken ternary phase particle. The detection of small precipitated particles by HRTEM is also impeded if the particles are located in thicker regions of the specimen. Figure 11b and c show ADF images of the same location obtained in STEM mode with different camera lengths. The HAADF image acquired with CL = 80 mm (Figure 11b) shows strong brighter contrast from bigger quasicrystal particles and weak bright contrast from the smaller precipitates. At the same time these smaller precipitates appear brighter than the matrix in HAADF, which indicates that these precipitates are binary phase consisting of Mg and Zn atoms. In an LAADF image acquired at higher camera length of 560 mm (Figure 11c), the smaller precipitate marked with a circle is apparently directly attached to the bigger particle. A few neighboring precipitates also exhibit similarly strong bright contrast. Thus HAADF/LAADF imaging together indicates that the particle could be newly heterogeneously precipitated particles from matrix formed at particle–matrix interfaces as well as inside the matrix during deformation. These new particles can further stabilize the grain refinement process because of creation of additional phase boundaries which can retard the grain growth process. In order to further confirm that this particle is precipitated, EDS line profile mapping in STEM mode was carried out. The composition profile shown in Figure 11d across the interface between ternary phase particle and the attached phase shows that there is sudden decrease in yttrium-L profile at the interface when it crosses the ternary phase particle, whereas Mg-K and Zn-K line profiles show almost no change in composition across the interface, which clearly indicates that the attached phase is a Mg-Zn binary phase.

This study establishes the grain size evolution by HPT processing. The grain size of the starting specimen (before deformation) was about 10 μm. The grain size after compression (N=0) was also similar to that of the initial sample, with formation of twins. After N=1/4 turns, the specimen shows two types of grains – one is subgrains with low angle boundaries, with size in the range of 300 nm to 2 μm. Another type of grains is recrystallized grains of size from 80 to 170 nm. The recrystallization is complete after N=5 turns of HPT, with an average size of about 150 nm. As the strain increases, more subgrains are formed, but the recrystallized grain sizes remains nearly the same.

The microstructural evolution during HPT process as studied here shows similarities to the binary Mg-3.4at%Zn alloy studied by HPT, where the initial alloy was extruded (and consequently had a texture) and subsequently solution treated at 573 K, to obtain a grain size of about 28 μm [16,17]. In the binary Mg-Zn alloy, heavy twinning of matrix grains is observed initially, followed by subgrain formation. At N=3, a bimodal microstructure is reported, of large grains with accumulated strain and fine recrystallized grains. Zinc segregation and consequent precipitation occurred at the grain boundaries, cited as the reason for very fine grain size by pinning of the boundaries, resulting in the final grain size of about 140 nm [16]. In the present study of the ternary Mg-Zn-Y alloy, a very small amount of zinc was available in the matrix, due to formation of the quasicrystal phase with a general composition Mg${}_{3}$Zn${}_{6}$Y. However, zinc segregation at the boundaries is still observed. The source of zinc is most likely the supersaturation of the matrix from the solution treatment, and a possible decomposition of the ternary phase under strain. Details of chemical compositional analysis will be published in a separate paper. A complete recrystallization is observed by N=5, assisted by recrystallization on the quasicrystal phase particles.

## 4. Conclusions

Microstructural evolution, especially grain refinement by recrystallization during severe plastic deformation of a Mg-3Zn-0.5Y (at%) alloy processed by HPT at room temperature was studied by conventional TEM as well as STEM-low angle annular dark field (LAADF) imaging technique with an optimized annular detection angle. Suitability of LAADF for studying severe plastically deformed alloys is demonstrated, where grain boundaries can be imaged even in highly strained regions. The following are the main findings of this study.

(1) Extensive twinning was observed due to compression in the N=0 sample. Specimen tilt-dependent channeling effect studies demonstrate that sources of contrast from the twin are not same in HAADF and LAADF imaging modes. Observation of twins is sensitive to sample tilt due to channeling effects, while strain contrast is not.
(2) No recrystallization nucleation is observed in twins.
(3) LAADF imaging alone permits a clear view of initial grain fragmentation and formation of sub grain structures in the N=1/4 specimen.
(4) By LAADF imaging, nucleation of recrystallization was detected at an early deformation stage of N=1/4. Severe strain contrast effects were subdued to observe the grain boundaries.
(5) Complete recrystallization was observed in the N=5 sample. Clear observation of grain boundaries became possible in LAADF by eliminating strong strain contrast within grains and by reduction of the effect of uneven specimen thickness. Besides, by HAADF contrast, particles of second phase were clearly distinguished from the matrix grains. Average grain size in the N=5 specimen was determined to be 150±25 nm.
6. Nano-sized binary phase precipitates were analyzed by ADF imaging and composition profile, and distinguished from the fragments of the ternary phase.
